# Supplementary material for: Recombinant Art v4.01 protein produces immunological tolerance by subcutaneous immunotherapy in a wormwood pollen-driven allergic asthma female mouse model
Source: PLoS One. 2024 Jun 28;19(6):e0280418. doi: 10.1371/journal.pone.0280418 (PMC11213334; doi:10.1371/journal.pone.0280418)
Supplement: S1 Fig — A Epitopes of Art v4.01 were highlighted on the amino acid sequence. B, C: B-cell and T-cell epitope positions identified on the surface of the 3D structure of Art v4.01. (DOCX) [file pone.0280418.s001.docx]

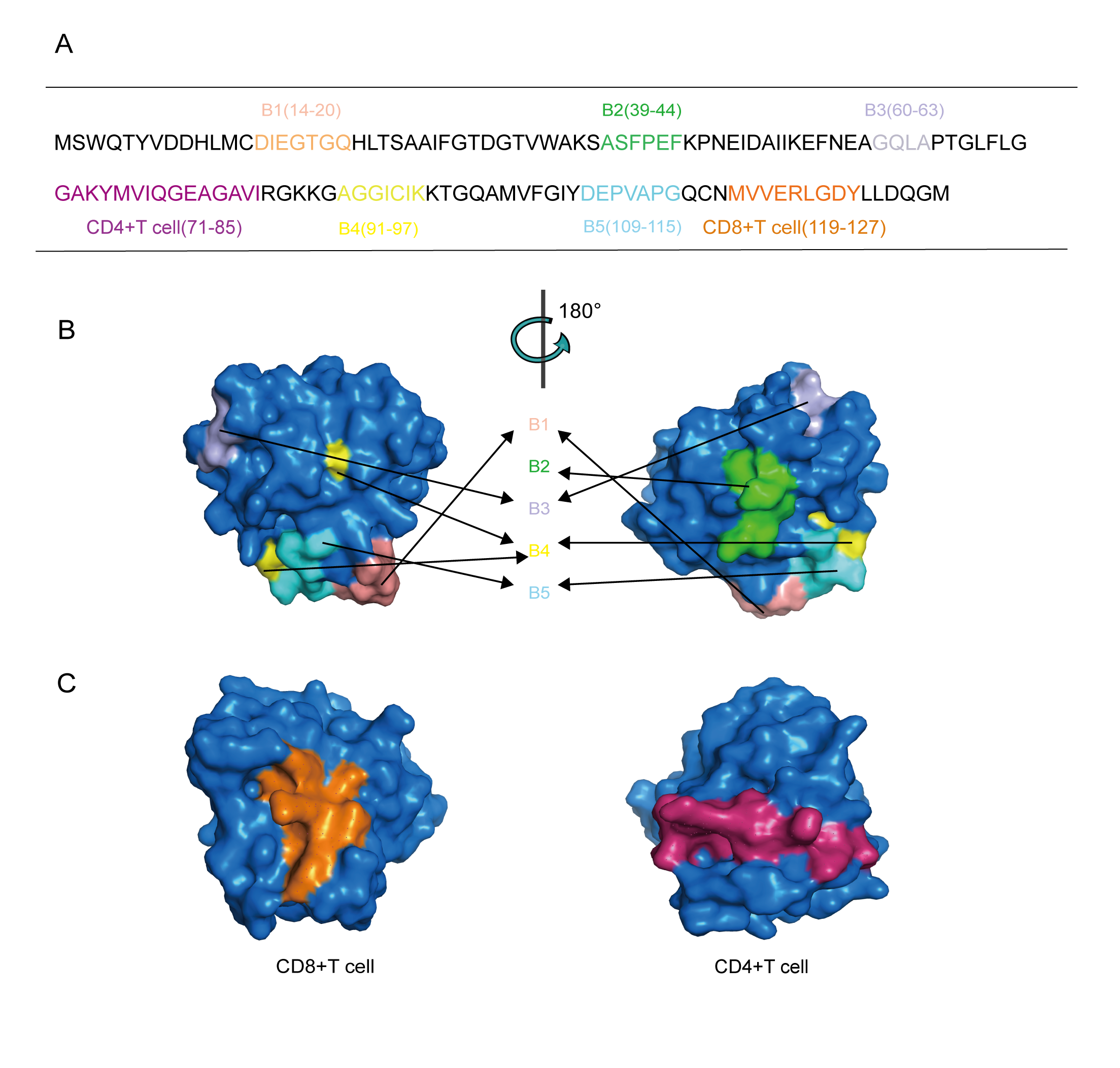


**Fig S1.** **Surface representation of predicted B- and T-cell epitopes of Art v4.01.** **A** Epitopes of Art v4.01 were highlighted on the amino acid sequence. **B, C:** B-cell and T-cell epitope positions identified on the surface of the 3D structure of Art v4.01. **B** B1-B5 are the predicted B-cell epitopes. **C** The left image is the epitope of CD8+T cell and the right is the epitope of CD4+T cell.
