# Supplementary material for: Recombinant Art v4.01 protein produces immunological tolerance by subcutaneous immunotherapy in a wormwood pollen-driven allergic asthma female mouse model
Source: PLoS One. 2024 Jun 28;19(6):e0280418. doi: 10.1371/journal.pone.0280418 (PMC11213334; doi:10.1371/journal.pone.0280418)
Supplement: S2 Fig — (DOCX) [file pone.0280418.s002.docx]

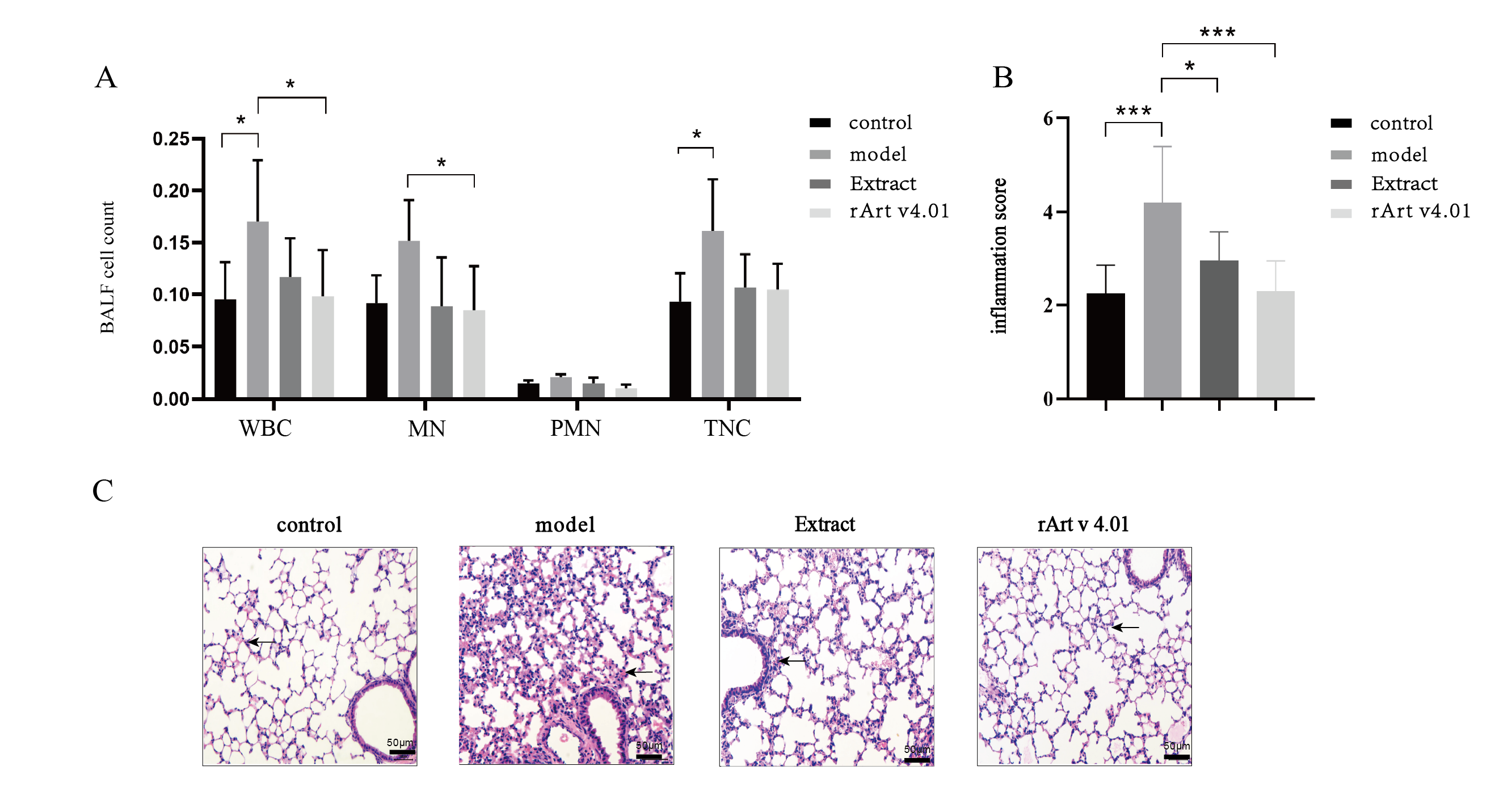


**Fig S2.** **These data are the result of repeated experiments. A** **Comparison of BALF cell counts between SCIT treatment groups.** **B** **The inflammation scores in lung histology.** The scores were analyzed by one-way analysis of variance with Tukey's multiple comparison test. **C** **H&E staining of the lung tissues.** Scale bar = 50 μm. Arrows point to the inflammatory cell infiltrate. Data represent the mean ± SD. *, *P* < 0.05; ***, *P* < 0.001.
