# Supplementary material for: Recombinant Art v4.01 protein produces immunological tolerance by subcutaneous immunotherapy in a wormwood pollen-driven allergic asthma female mouse model
Source: PLoS One. 2024 Jun 28;19(6):e0280418. doi: 10.1371/journal.pone.0280418 (PMC11213334; doi:10.1371/journal.pone.0280418)
Supplement: S3 Fig — (DOCX) [file pone.0280418.s003.docx]

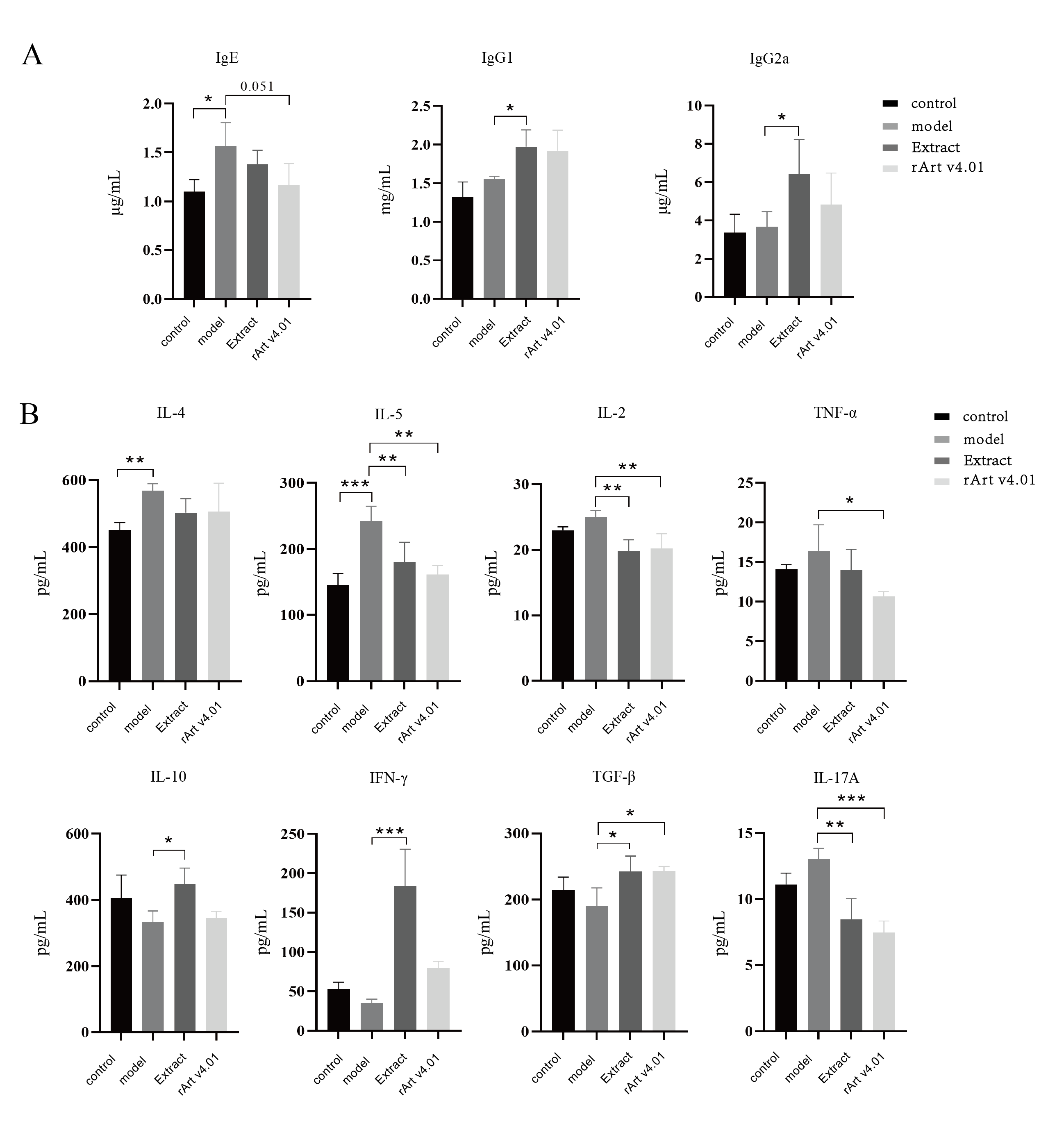
**Fig S3.** **These data are the result of repeated experiments. A Quantification of total serum IgE, IgG1, and IgG2a levels** in short-term treatment groups of BALB/c mice. **B** **Quantification of the levels of cytokines**, such as IL-4, IL-5, IL-2, IL-17A, IL-10, TNF-α, IFN-γ, and TGF-β were analyzed by ELISA in spleen homogenate in short-term treatment groups of BALB/c mice. Data represent the mean ± SD. *, *P* < 0.05; **, *P* < 0.01, ***, *P* < 0.001.
