# Supplementary material for: Recombinant Art v4.01 protein produces immunological tolerance by subcutaneous immunotherapy in a wormwood pollen-driven allergic asthma female mouse model
Source: PLoS One. 2024 Jun 28;19(6):e0280418. doi: 10.1371/journal.pone.0280418 (PMC11213334; doi:10.1371/journal.pone.0280418)
Supplement: S4 Fig — (DOCX) [file pone.0280418.s004.docx]

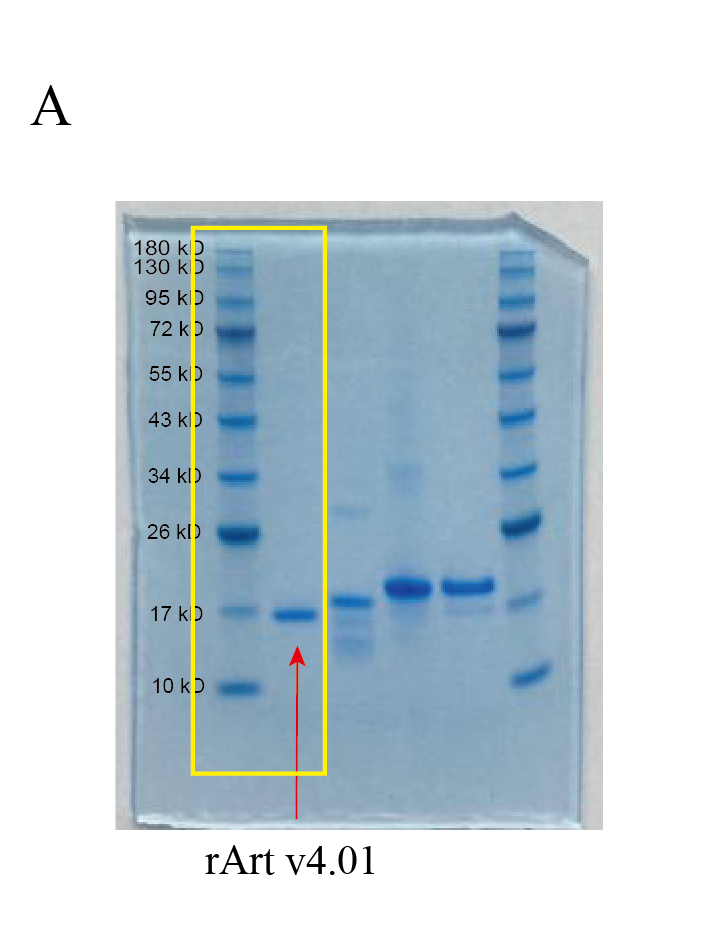


Fig S4. The original underlying images of SDS-PAGE analysis for rArt v4.01 in Fig 1C, the target bands have been marked with red arrows. The target bands were marked with red arrows. Figure panel were marked with yellow frame which was generated from that original image.
