# Supplementary material for: Recombinant Art v4.01 protein produces immunological tolerance by subcutaneous immunotherapy in a wormwood pollen-driven allergic asthma female mouse model
Source: PLoS One. 2024 Jun 28;19(6):e0280418. doi: 10.1371/journal.pone.0280418 (PMC11213334; doi:10.1371/journal.pone.0280418)
Supplement: S5 Fig — (DOCX) [file pone.0280418.s005.docx]

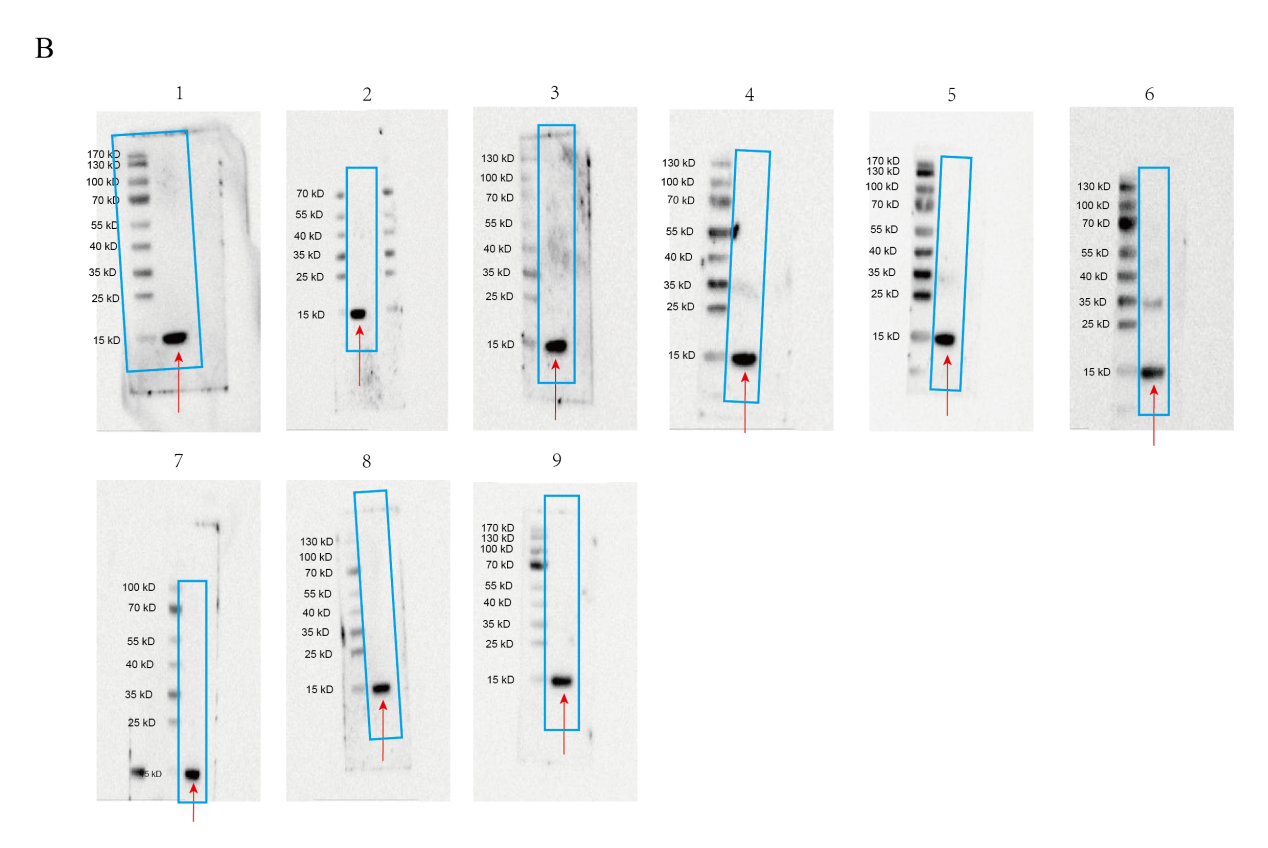


Fig S5. The original underlying images of Western blotting for rArt v4.01 with serum from patients in Fig 1D. The numbers (1-9) at the top of the raw blots correspond to the images of 9 allergic patients in the manuscript. These images was made by chemiluminescence (Thermo Fisher) using a ChemiDoc image analysis system (BioRAD,Hercules, CA, USA).The target bands were marked with red arrows. Figure panel were marked with blue frame which was generated from that original image.
