# Supplementary material for: Recombinant Art v4.01 protein produces immunological tolerance by subcutaneous immunotherapy in a wormwood pollen-driven allergic asthma female mouse model
Source: PLoS One. 2024 Jun 28;19(6):e0280418. doi: 10.1371/journal.pone.0280418 (PMC11213334; doi:10.1371/journal.pone.0280418)
Supplement: S1 Table — (DOCX) [file pone.0280418.s006.docx]

| **Table S1.** Prediction results of B-cell epitopes of Art v4.01 | | | |
| --- | --- | --- | --- |
| Peptide | Sequence | Position | Tools |
| B1 | DIEGTGQ | 14-20 | ABCpred, IEDB-AR |
| B2 | ASFPEF | 39-44 | ABCpred, IEDB-AR, BPAP |
| B3 | GQLA | 60-63 | ABCpred, IEDB-AR, BPAP |
| B4 | AGGICIK | 91-97 | ABCpred, BPAP |
| B5 | DEPVAPG | 109-115 | ABCpred, IEDB-AR, BPAP |
