# Supplementary material for: Recombinant Art v4.01 protein produces immunological tolerance by subcutaneous immunotherapy in a wormwood pollen-driven allergic asthma female mouse model
Source: PLoS One. 2024 Jun 28;19(6):e0280418. doi: 10.1371/journal.pone.0280418 (PMC11213334; doi:10.1371/journal.pone.0280418)
Supplement: S2 Table — (DOCX) [file pone.0280418.s007.docx]

| **Table S2.** Prediction results of CD4+ T and CD8+ T cell epitopes of Art v4.01 | | | |
| --- | --- | --- | --- |
| Peptide | Sequence | Position | Tools |
| CD4+T cell | GAKYMVIQGEAGAVI | 71-85 | NetMHCII, NetMHC II pan, SYFPEITHI, IEBD |
| CD8+T cell | MVVERLGDY | 119-127 | NetMHC, SYFPEITHI, IEBD |
