# Supplementary material for: Recombinant Art v4.01 protein produces immunological tolerance by subcutaneous immunotherapy in a wormwood pollen-driven allergic asthma female mouse model
Source: PLoS One. 2024 Jun 28;19(6):e0280418. doi: 10.1371/journal.pone.0280418 (PMC11213334; doi:10.1371/journal.pone.0280418)
Supplement: S1 Raw image — (PDF) [file pone.0280418.s008.pdf]

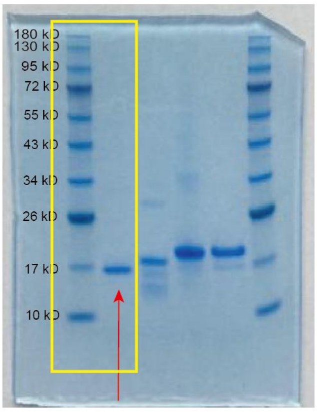

rArt v4.01

Fig S2. The original underlying images of SDS-PAGE analysis for rArt v4.01 in Fig 1C, the target bands have been marked with red arrows. The target bands were marked with red arrows. Figure panel were marked with yellow frame which was generated from that original image.

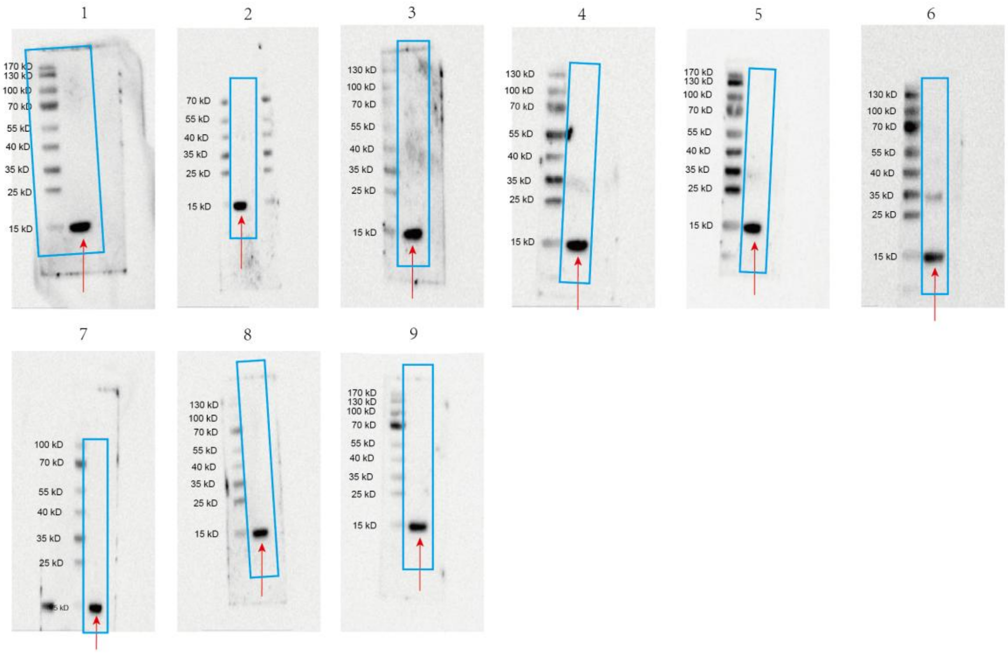

Fig S3. The original underlying images of Western blotting for rArt v4.01 with serum from patients in Fig 1D. The numbers (1-9) at the top of the raw blots correspond to the images of 9 allergic patients in the manuscript. These images was made by chemiluminescence (Thermo Fisher) using a ChemiDoc image analysis system (BioRAD,Hercules, CA, USA).The target bands were marked with red arrows. Figure panel were marked with blue frame which was generated from that original image.
