# Supplementary material for: Recombinant Art v4.01 protein produces immunological tolerance by subcutaneous immunotherapy in a wormwood pollen-driven allergic asthma female mouse model
Source: PLoS One. 2024 Jun 28;19(6):e0280418. doi: 10.1371/journal.pone.0280418 (PMC11213334; doi:10.1371/journal.pone.0280418)
Supplement: Attachment — Submitted filename: Response to Reviewers5.28.docx [file pone.0280418.s009.docx]

**Dear Editor**

Thank you for your letter and for the reviewers’ comments concerning our manuscript entitled “Recombinant Art v4.01 Protein Produces Immunological Tolerance by Subcutaneous Immunotherapy in a Wormwood Pollen-driven Allergic Asthma Mouse Model” . Those comments were greatly valuable and significant for revising and improving this manuscript. We have carefully studied the reviewers’ comments, and we have accordingly applied revisions. All the changes have been highlighted in red in the revised version of the manuscript, and we hope that the revised version of the manuscript would be acceptable for publication in your prestigious journal. The reviewers’ comments have been replied one-by-one as follows:

**#** Journal Requirements

When submitting your revision, we need you to address these additional requirements.

1. Please ensure that your manuscript meets PLOS ONE's style requirements, including those for file naming.

**Response:** The manuscript was modified according to the PLOS ONE’s formatting rules, and all the changes have been highlighted in red in the revised version of the manuscript.

2. To comply with PLOS ONE submissions requirements, in your Methods section, please provide additional information regarding the experiments involving animals and ensure you have included details on (1) methods of sacrifice, (2) methods of anesthesia and/or analgesia, and (3) efforts to alleviate suffering.

**Response:** We described the methods of sacrifice was cervical dislocation on line 163, the methods of anesthesia was used 4.5% isoflurane on line 148, and the methods of alleviating suffering was anesthetized mice on line 167.

3. Please state what role the funders took in the study.

**Response:** The funders play a major role in study design, decision to publish, and revision of the manuscript. And this amended role of funder statement in cover letter.

4. PLOS requires an ORCID iD for the corresponding author in Editorial Manager on papers submitted after December 6th, 2016.

**Response:** The corresponding author’s ORCID iD was already associated with his account of PLOS ONE.

5. PLOS ONE now requires that authors provide the original uncropped and unadjusted images underlying all blot or gel results reported in a submission’s figures or Supporting Information files.

**Response:** We provided the original underlying images for all blot and gel data reported in supporting information files, and described the original underlying images were placed in the supporting information files in cover letter.

6. Please review your reference list to ensure that it is complete and correct. If you have cited papers that have been retracted, please include the rationale for doing so in the manuscript text, or remove these references and replace them with relevant current references.

**Response:** The references had been corrected which no papers that have been retracted.

**# Reviewer 1**

Congratulation on your great work. The article appears scientifically sound, with an explicit method and outcome.

1. I got confused with tagged figures in the result and discussion. Namely, I couldn't find figures (C2, 3B, and 5B). Kindly review the supplement figures.

**Response:**

1)Fig 2C represents the inflammation scores in lung histology which was in the middle part of the second image on lines 222 and 247.

2)Fig. 3B represents the quantification of the levels of cytokines, IL-4, IL-5, IL-2, IL-17A , IL-10, TNF-α, IFN-γ and TGF-β analysed by ELISA in spleen homogenate in short-term treatment groups of BALB/c mice studieds which was in the bottom part of the 3rd image on lines 228-230, 259 and 262.

.

3)We were very sorry, Fig. 5B on line 244 was an author’s error and should be corrected to Fig. 2B, which represents the comparison of BALF cell counts between SCIT treatment groups.

4) Supplemental Fig. 2 was corrected to S1 Fig. and Supplemental Tab. 2，3 was corrected to S1 Table and S2 Table on line 212.

**# Reviewer 2**

1. The authors were trying to investigate the ability of the recombinant Art v4.01 to produce immunological tolerance in BALB/c mice model of allergic asthma in comparison to the use of crude wormwood extracts. However, the sample size (n=8) is not enough to achieve the main objective of this study.

**Response:** Thanks for your significant comment. I agree with your view which the sample size (n=8) was not enough to achieve the main objective of this study. Meanwhile, we determined the number of mice based on the available literature, and the number of mice in their study was 8, 7, 6, and 6^[1-4]^. Therefore, I think that the results of this study could provide support for the main objective of this study, however, if the main objective of this study was to be achieved, more mice need to be performed to get more convincing results.

[1] Hesse L, van Ieperen N, Habraken C, Petersen AH, Korn S, Smilda T, et al. Subcutaneous immunotherapy with purified Der p1 and 2 suppresses type 2 immunity in a murine asthma model. Allergy. 2018;73(4):862-874.

[2] Heldner A, Alessandrini F, Russkamp D, Heine S, Schnautz B, Chaker A, et al. Immunological effects of adjuvanted low-dose allergoid allergen-specific immunotherapy in experimental murine house dust mite allergy. Allergy. 2022;77(3):907-919.

[3] Liu J, Yin J. Immunotherapy With Recombinant Alt a 1 Suppresses Allergic Asthma and Influences T Follicular Cells and Regulatory B Cells in Mice. Front Immunol. 2021;12:747730.

[4] Tabynov K, Babayeva M, Nurpeisov T, Fomin G, Nurpeisov T, Saltabayeva U, et al. Evaluation of a Novel Adjuvanted Vaccine for Ultrashort Regimen Therapy of Artemisia Pollen-Induced Allergic Bronchial Asthma in a Mouse Model. Front Immunol. 2022;13:828690.

2. The manuscript needs extensive revision for language and grammar (Revision by a professional is recommended).

**Response:** We had carefully revised our manuscript, which was also polished by a native English speaker from a language services company.

3. In the materials and methods section, the groups of animals and different treatments should be identified more clearly. Furthermore, the whole section should be rephrased to avoid confusion.

**Response:** In order to more clearly identify the groups of animals and different treatments, negative control group and NC group was corrected to control group on lines 144, 149, 161, 215, 218, 221, 225 and 228-230; Positive control group and PC group was corrected to model group on lines 33, 144,161, 216, 218, 221-222, 224, 227, 242, 246, 250, 253, 255, 259; rArt v4.01 SCIT group abbreviated as rArt v4.01; wormwood pollen extract SCIT group abbreviated as Extract. sensitized subcutaneously was corrected to sensitized via subcutaneous injection on line 144; intranasal instillation was corrected to intranasal inoculation on lines 148, 162; ‘Mice were subcutaneously injected’ was corrected to ‘SCIT was carried out’ on line 160.

4. IgE binding capacity should be identified

**Response:**

IgE binding capacity of rArt v 4 to wormwood pollen specific IgE was examined using sera of the model group of mice based on Western blot and enzyme-linked immunosorbent reaction (ELISA).

First of all, Western blot was performed using sera from wormwood pollen-allergic mice. The results showed that rArt v 4 was able to bind to IgE epitopes of allergic samples (Fig 1). It showed IgE reactivity in the serum from wormwood pollen-allergic mice.

Fig 1. IgE binding to wormwood pollen extracts was investigated by immunoblot. Band around 35kD was found to exist as homodimers of recombinant Art v 4.

In addition, Indirect-ELISA was performed to determine sIgE against rArt v 4 using sera from wormwood pollen-allergic mice. Plates were coated with rArt v 4 (5ug/ml) in ELISA Binding Buffer (ScyTek laboratories, USA) and kept at 4℃ overnight. After washing three times with phosphate-buffered saline, plates were blocked with Super Block (ScyTek laboratories, USA) for 10 minutes. Then, plates were incubated for 2 hours with sera from wormwood pollen-allergic mice and blank control mice at 1:0, 1:1, 1:2, 1:4, 1:8, 1:16, 1:32, 1:64, 1:128 and 1:256 dilution, respectively. After washing, the goat anti-mouse IgE-HRP at 1:2000 dilution was added to the wells. The color was developed using TMB Soluble Reagent (ScyTek laboratories, USA) , and then absorbance at 450nm was measured. The results showed that sera from wormwood pollen-allergic mice could bind with rArt v 4 and (Fig 2) and the binding of sIgE to rArt v 4 is weakened as the sera dilution increase.

Fig 2. IgE binding to wormwood pollen extracts was investigated by ELISA

5. Many words are splitting at the end of lines

**Response:** The formatting of manuscript had been changed which words could not be split at the end of lines.
