# Supplementary material for: Recombinant Art v4.01 protein produces immunological tolerance by subcutaneous immunotherapy in a wormwood pollen-driven allergic asthma female mouse model
Source: PLoS One. 2024 Jun 28;19(6):e0280418. doi: 10.1371/journal.pone.0280418 (PMC11213334; doi:10.1371/journal.pone.0280418)
Supplement: Attachment — Submitted filename: Response to Reviewers9.14.docx [file pone.0280418.s010.docx]

**Dear Editor**

Thank you for your letter and for the reviewers’ comments concerning our manuscript entitled “Recombinant Art v4.01 protein produces immunological tolerance by subcutaneous immunotherapy in a wormwood pollen-driven allergic asthma female mouse model” . Those comments were greatly valuable and significant for revising and improving this manuscript. We have carefully studied the reviewers’ comments, and we have accordingly applied revisions. All the changes have been highlighted in red in the revised version of the manuscript, and we hope that the revised version of the manuscript would be acceptable for publication in your prestigious journal. The reviewers’ comments have been replied one-by-one as follows:

**# Journal Requirements**

1. The number of animals per group used is very small and the experiment was not independently repeated in a new cohort of animals to validate the results. There is also an issue of the selective female mouse usage. When study is performed in one sex of animals, this has to be noted in the title and abstract, and the discussion need to address this limitation stating that the results in males could be different and should be further investigated in the future studies.

**Response:** These questions will be responded to in detail in the response to reviewers.

**# Reviewer 1**

1. Dear Authors. congratulation for your work. You get respond to the comment and appeal my confusion.
   wish you all the best

**Response:** Dear reviewer, thank you very much for your comments , which were greatly valuable and significant for revising and improving this manuscript.

**# Reviewer 2**

1. The authors have poorly responded to some of the comments including the number of mice subjects which is one of the most important comments.

**Response:** I am very sorry that I did not provide good feedback in response to the last comment. Insufficient number of mice is one of the limitations of this study and we will mention this limitation in the discussion section on lines 326-328. However, as stated in the last reply letter, the number of mice used in this study was formulated based on the literature of excellent journals in the field of this study, e.g. Allery, Frontiers in immunology, therefore, the results of this study could provide support for the main objective of this study. In addition, this animal model is stable and reliable, therefore, a small number of mice were used for the experiments in this study to meet animal welfare requirements. The reason why this animal model is stable and reliable are that the animal sensitization model and specific immunotherapy were designed, as previously described [1-3]. In addition, the results of this study can also support this view.

2. Furthermore, the authors should justify their selection of female over male mice otherwise the experiment should use both male and female mice to exclude the gender effect. There should also be replication of the animal experiment to ensure fidelity.

**Response:** Numerous studies indicate that female BALB/c mice are more susceptible to the development of allergic inflammation than male mice. For example, allergens-driven accumulation of neutrophils, eosinophils and macrophages were significantly higher in females compared to males ^[4]^. group 2 innate lymphoid cells (ILC2s)-activating cytokines including IL-33, IL-7 and TSLP were more highly expressed in whole lung homogenate samples prepared from naive post pubertal female mouse lung than male mouse lung ^[5]^. Female compared with male mice generally developed more pronounced antibody and inflammatory responses ^[6-9]^. However, testosterone, signaling through the androgen receptor (AR), decreased Th2-mediated allergic inflammation and type 2 innate immune responses during allergic inflammation ^[10]^. And androgen signaling suppresses cytokine production of Th2 cells by inducing DUSP-2 ^[11]^.Therefore, female BALB/c mice were used in this study. The use of female mice in this study has been mentioned in the title and abstract on lines 3, 23, and the address this limitation has been noted in the discussion section on lines 328-329 , as well as noting that the results may be different for male mice on lines 329-330 and stating that gender effects will be further investigated in future studies on lines 330-331.

Then, the experiment was not independently repeated is one of the limitations of this study, future research will remedy this limitation.

1. Liu J, Yin J. Immunotherapy With Recombinant Alt a 1 Suppresses Allergic Asthma and Influences T Follicular Cells and Regulatory B Cells in Mice. Front Immunol. 2021;12:747730. https://doi.org/10.3389/fimmu.2021.747730 PMID: 34804031.

2. Zhang Q, Xi G, Yin J. Artemisia sieversiana pollen allergy and immunotherapy in mice. Am J Transl Res. 2021;13(12):13654-13664. PMID: 35035704.

3. Hesse L, Petersen AH, Nawijn MC. Methods for Experimental Allergen Immunotherapy: Subcutaneous and Sublingual Desensitization in Mouse Models of Allergic Asthma. Methods Mol Biol. 2021;2223:295-335. https://doi.org/10.1007/978-1-0716-1001-5_20 PMID: 33226602.

4. Mostafa D, Hemshekhar M, Piyadasa H, Altieri A, Halayko AJ, Pascoe CD, et al. Characterization of sex-related differences in allergen house dust mite-challenged airway inflammation, in two different strains of mice. Sci Rep. 2022;12(1):20837. https://doi.org/10.1038/s41598-022-25327-7 PMID: 36460835.

5. Matha L, Shim H, Steer CA, Yin YH, Martinez-Gonzalez I, Takei F. Female and male mouse lung group 2 innate lymphoid cells differ in gene expression profiles and cytokine production. PLoS One. 2019;14(3):e0214286. https://doi.org/10.1371/journal.pone.0214286 PMID: 30913260.

6. Alberg T, Hansen JS, Lovik M, Nygaard UC. Particles influence allergic responses in mice--role of gender and particle size. J Toxicol Environ Health A. 2014;77(5):281-92. https://doi.org/10.1080/15287394.2013.863746 PMID: 24588227.

7. Hansen JS, Alberg T, Rasmussen H, Lovik M, Nygaard UC. Determinants of experimental allergic responses: interactions between allergen dose, sex and age. Scand J Immunol. 2011;73(6):554-67. https://doi.org/10.1111/j.1365-3083.2011.02529.x PMID: 21323693.

8. Okuyama K, Wada K, Chihara J, Takayanagi M, Ohno I. Sex-related splenocyte function in a murine model of allergic asthma. Clin Exp Allergy. 2008;38(7):1212-9. https://doi.org/10.1111/j.1365-2222.2008.03015.x PMID: 18498415.

9. Melgert BN, Postma DS, Kuipers I, Geerlings M, Luinge MA, van der Strate BW, et al. Female mice are more susceptible to the development of allergic airway inflammation than male mice. Clin Exp Allergy. 2005;35(11):1496-503. https://doi.org/10.1111/j.1365-2222.2005.02362.x PMID: 16297148.

10. Fuseini H, Yung JA, Cephus JY, Zhang J, Goleniewska K, Polosukhin VV, et al. Testosterone Decreases House Dust Mite-Induced Type 2 and IL-17A-Mediated Airway Inflammation. J Immunol. 2018;201(7):1843-1854. https://doi.org/10.4049/jimmunol.1800293 PMID: 30127088.

11. Ejima A, Abe S, Shimba A, Sato S, Uehata T, Tani-Ichi S, et al. Androgens Alleviate Allergic Airway Inflammation by Suppressing Cytokine Production in Th2 Cells. J Immunol. 2022;209(6):1083-1094. https://doi.org/10.4049/jimmunol.2200294 PMID: 35977797.
