# Supplementary material for: Recombinant Art v4.01 protein produces immunological tolerance by subcutaneous immunotherapy in a wormwood pollen-driven allergic asthma female mouse model
Source: PLoS One. 2024 Jun 28;19(6):e0280418. doi: 10.1371/journal.pone.0280418 (PMC11213334; doi:10.1371/journal.pone.0280418)
Supplement: Attachment — Submitted filename: Response to Reviewers.docx [file pone.0280418.s011.docx]

**Dear Editor**

Thank you for comments concerning our manuscript entitled “Recombinant Art v4.01 protein produces immunological tolerance by subcutaneous immunotherapy in a wormwood pollen-driven allergic asthma female mouse model”. Those comments were greatly valuable and significant for revising and improving this manuscript. In order to ensure rigor and reproducibility of this experiment, we repeated the most important readouts to ensure that the therapy works consistently. The data of the repeated experiment showed the same trend with the results of the previous experiment, indicating that the results of this experiment are credible and repeatable. Thus, the new data were shown in the supplement material. All the changes have been highlighted in red in the revised version of the manuscript, and we hope that the revised version of the manuscript would be acceptable for publication in your prestigious journal. The new data have been shown as follows:

Compared with the model group, the number of WBCs, TNCs, MNs, and PMNs in the BALF in the treatment group showed a downward trend, in which the number of WBCs, MNs in the rArt v4.01 group significantly decreased (P < 0.05, Fig. 1A), and the number of inflammatory cells in the extract group did not significantly vary (P > 0.05, Fig. 1A). The inflammation scores in the rArt v4.01 and extract groups [2.30±0.64 and 2.95±0.61, respectively] were significantly lower than those in the model group (4.20±1.19, P < 0.05, Fig. 1B). This part of the result was similar to the previous experimental results. It was demonstrated that both strategies mitigated allergic inflammation, including a decrease in the number of inflammatory cells in BALF, and reduced congestion and inflammatory cell infiltration in the alveolar wall. However, the ability of rArt v4.01 to inhibit inflammation was superior to that of wormwood.

In addition, serum IgE level in the rArt v4.01 group was lower than that in the model group (1.17±0.18) μg/ml (P = 0.051), while the extract group had no significant effect (P > 0.05, Fig. 2A). In contrast, serum IgG1 levels in the extract groups were 1.97±0.19 mg/ml, which were significantly higher than those in the model group (1.56±0.03 mg/ml, P < 0.05, Fig. 2A). Similar to IgG1, serum IgG2a level was upregulated in the SCIT group. Importantly, serum IgG2a level was significantly higher in the extract group than that in the model group (6.44±1.55 vs. 3.68±0.68 µg/ml) (P < 0.05), while rArt v4.01 had no significant effect (P > 0.05, Fig. 2A). This part of the result was also similar to the previous experimental results. Only recombinant protein treatment significantly reduced serum IgE level. And only the wormwood was effective in inducing serum levels of IgG1 and IgG2a, while rArt v4.01 did not produce similar results.

The levels of Th2-related cytokines (IL-5 and IL-2) and Th17-related cytokines(IL-17A) were significantly downregulated in the rArt v4.01 and extract group compared with those in the model group (P < 0.05, Fig. 2B), with a greater decrease in the rArt v4.01 group compared to the extract group. TNF-α was significantly downregulated in the rArt v4.01 group compared with the model group (P < 0.05, Fig. 2B). In contrast, the levels of Treg-related cytokine (IL-10) and Th1-type cytokine (IFN-γ) were significantly upregulated in the extract group (P < 0.05), TGF-β significantly upregulated in the rArt v4.01 and extract group compared with those in the model group (P < 0.05, Fig. 2B). In addition, the levels of IL-4 did not significantly differ between the two treatment groups (P > 0.05, Fig. 2B). This part of the result was also similar to the previous experimental results. It was also confirmed that rArt v4.01 inhibited Th2 and Th17 response more strongly than wormwood, while induced Th1, regulatory T cells (Tregs) weaker than crude extract.

**Fig 1.** **A** **Comparison of BALF cell counts between SCIT treatment groups.** **B** **The inflammation scores in lung histology.** The scores were analyzed by one-way analysis of variance with Tukey's multiple comparison test. **C** **H&E staining of the lung tissues.** Scale bar = 50 μm. Arrows point to the inflammatory cell infiltrate. Data represent the mean ± SD. *, *P* < 0.05; ***, *P* < 0.001.

**Fig 2.** **A Quantification of total serum IgE, IgG1, and IgG2a levels** in short-term treatment groups of BALB/c mice. **B** **Quantification of the levels of cytokines**, such as IL-4, IL-5, IL-2, IL-17A, IL-10, TNF-α, IFN-γ, and TGF-β were analyzed by ELISA in spleen homogenate in short-term treatment groups of BALB/c mice. Data represent the mean ± SD. *, *P* < 0.05; **, *P* < 0.01, ***, *P* < 0.001.
