# Supplementary material for: Recombinant Art v4.01 protein produces immunological tolerance by subcutaneous immunotherapy in a wormwood pollen-driven allergic asthma female mouse model
Source: PLoS One. 2024 Jun 28;19(6):e0280418. doi: 10.1371/journal.pone.0280418 (PMC11213334; doi:10.1371/journal.pone.0280418)
Supplement: Attachment — Submitted filename: Response to Reviewers3.10.docx [file pone.0280418.s012.docx]

**Dear Editor**

Thank you for comments concerning our manuscript entitled “Recombinant Art v4.01 protein produces immunological tolerance by subcutaneous immunotherapy in a wormwood pollen-driven allergic asthma female mouse model”. Those comments were greatly valuable and significant for revising and improving this manuscript.

Firstly, the new supplementary data have been cited in the manuscript on lines 251-254, 256-258, 268-273, 278-286, 318-322 and324-328. Secondly, we include following note in the figure legend. Replicates of these experiments are shown in Fig S4, 5 (lines 160 and 241). Thirdly, The independent repeats have been mentioned in the method section on lines 202-204. Finally, All the changes have been highlighted in red in the revised version of the manuscript, and we hope that the revised version of the manuscript would be acceptable for publication in your prestigious journal.
